# Core−Shell Molecularly Imprinted Polymers on Magnetic Yeast for the Removal of Sulfamethoxazole from Water

**DOI:** 10.3390/polym12061385

**Published:** 2020-06-20

**Authors:** Liang Qiu, Guilaine Jaria, María Victoria Gil, Jundong Feng, Yaodong Dai, Valdemar I. Esteves, Marta Otero, Vânia Calisto

**Affiliations:** 1 Department of Materials Science and Technology, Nanjing University of Aeronautics & Astronautics, Nanjing 210016, China; Qiuliangyzl@126.com (L.Q.); jundongfeng@nuaa.edu.cn (J.F.); yd_dai@nuaa.edu.cn (Y.D.); 2 CESAM & Department of Chemistry, University of Aveiro, Campus Universitário de Santiago, 3810-193 Aveiro, Portugal; jaria.guilaine@ua.pt (G.J.); valdemar@ua.pt (V.I.E.); vania.calisto@ua.pt (V.C.); 3 Instituto de Ciencia y Tecnología del Carbono (INCAR-CSIC), C/ Francisco Pintado Fe 26, 33011 Oviedo, Spain; victoria.gil@incar.csic.es; 4 CESAM & Department of Environment and Planning, Campus Universitário de Santiago, 3810-193 Aveiro, Portugal

**Keywords:** antibiotics, emerging contaminants, pharmaceuticals, wastewater treatment, polymeric adsorbents, magnetization

## Abstract

In this work, magnetic yeast (MY) was produced through an in situ one-step method. Then, MY was used as the core and the antibiotic sulfamethoxazole (SMX) as the template to produce highly selective magnetic yeast-molecularly imprinted polymers (MY@MIPs). The physicochemical properties of MY@MIPs were assessed by Fourier-transform infrared spectroscopy (FT-IR), a vibrating sample magnetometer (VSM), X-ray diffraction (XRD), thermogravimetric analysis (TGA), specific surface area (*S*_BET_) determination, and scanning electron microscopy (SEM). Batch adsorption experiments were carried out to compare MY@MIPs with MY and MY@NIPs (magnetic yeast-molecularly imprinted polymers without template), with MY@MIPs showing a better performance in the removal of SMX from water. Adsorption of SMX onto MY@MIPs was described by the pseudo-second-order kinetic model and the Langmuir isotherm, with maximum adsorption capacities of 77 and 24 mg g^−1^ from ultrapure and wastewater, respectively. Furthermore, MY@MIPs displayed a highly selective adsorption toward SMX in the presence of other pharmaceuticals, namely diclofenac (DCF) and carbamazepine (CBZ). Finally, regeneration experiments showed that SMX adsorption decreased 21 and 34% after the first and second regeneration cycles, respectively. This work demonstrates that MY@MIPs are promising sorbent materials for the selective removal of SMX from wastewater.

## 1. Introduction

Antibiotics are intensively used as human and veterinary medicines for the treatment and prevention of infectious diseases [1]. Among them, sulfamethoxazole (SMX) is a sulfonamide bacteriostatic antibiotic that has been commonly used during the last 80 years to treat urinary tract infections due to its low cost and broad spectrum of activity to treat bacterial diseases [2,3]. However, the widespread and indiscriminate use of SMX, as of other antibiotics, constitutes a huge potential threat to human health and contaminates natural ecosystems by affecting aquatic and soil organisms [4,5]. Recently, SMX has been detected in effluents of sewage treatment plants (STP), and also in surface and groundwater [6,7]. Indeed, it is known that pharmaceuticals (including SMX) can reach the aquatic environment in their unchanged or transformed forms mainly through discharge of effluents from municipal STP [7]. According to the statistics, more than 20,000 tons of SMX enter the environment worldwide every year, resulting in concentrations that range from 0.001 to 5.0 µg L^−1^ in untreated or treated wastewater [8,9,10]. Therefore, the problem of environmental contamination by SMX is of great concern as pathogen resistance is highly documented and has been induced even by low levels of antibiotics [11].

To solve the above-mentioned problems, substantial research efforts have been directed worldwide to develop sustainable treatments for the removal of antibiotics, including SMX, from contaminated waters, such as membrane separation, adsorption processes, photocatalysis, and chemical oxidation [12]. Among these treatments, adsorption-based processes have been highlighted to be efficient, easy to implement and, furthermore, avoid the generation of transformation products [13,14,15]. However, the application of these processes is quite challenging due to the characteristic features of contaminated wastewaters, namely, large discharge flux, complex composition, and very low antibiotic concentrations [16]. Increasing the adsorbent specificity has been proposed as a strategy to address these challenges and improve the efficiency of the adsorptive removal of antibiotics from such complex matrices [17].

Molecularly imprinted technology (MIT) involves the creation of tailor-made selective binding sites in a polymeric matrix with memory of the shape, size, and functional groups of the template. Thus, molecularly imprinted polymers (MIPs) have become increasingly attractive as adsorbent materials due to their capacity to selectively bind specific targets and to their promising characteristics, such as low cost, easy synthesis, high stability to harsh chemical and physical conditions, and excellent reusability [18,19]. In recent years, MIPs, whose application of the extraction and analysis of organic contaminants in environmental water samples is well-established [20], have been successfully used for the adsorptive removal of pharmaceuticals, including antibiotics, from contaminated water [21,22,23,24]. In the specific case of SMX adsorption by MIPs, few works have been published, with most of them aiming at the analytic quantification of this antibiotic. For example, Qin et al. [5] used Fe_3_-O_4_-chitosan MIPs for SMX selective extraction and determination in aqueous samples, with the produced materials having attained a maximum adsorption capacity of 4.32 mg g^−1^. Zhao et al. [25] prepared core–shell MIPs on the surface of magnetic carbon nanotubes (MCNTs@MIP) for SMX, the resulting material having a maximum SMX adsorption capacity from aqueous solution of 864.9 µg g^−1^. However, to the best of our knowledge, the removal of SMX from complex wastewaters using MIPs has just been assessed by Valtech et al. [19]. Among the materials produced by these authors [18], those having the largest maximum adsorption capacity (6.5 × 10^−5^ mol g^−1^ (16.5 mg g^−1^)) performed similarly to a commercial activated carbon in terms of removal, but presented higher selectivity toward SMX in the presence of other pharmaceuticals and better regeneration ability.

Despite the above-mentioned advantages and applications, the preparation of MIPs by conventional MIT has two main drawbacks: (1) The imprinted polymer matrices are thick and, thus, hold a small number of recognition sites per unit volume; and (2) the template molecules are deeply embedded in the matrix, so there is a diffusion barrier for them, the mass transfer rate is low, and binding to the recognition sites is somehow hampered [26]. Surface molecular imprinting has been proved to improve mass transfer, recognition, and binding ability relative to MIT [27]. Among solid-support substrates used for the surface molecular imprinting process, microbial nano-magnetic materials are alternative supporters that have many advantages compared to inorganic materials [28]: (1) They are easy to obtain and short generations can be artificially cultured [29]; (2) there are many surface chemical functional groups and so modification steps can be avoided, reducing secondary pollution; (3) cells can guide the regulation of the growth process of inorganic materials [30]; (4) microbial cells have a variety of structures and can provide a rich array of templates for nanomaterials by template-assisted synthesis; and (5) magnetic properties allow for a simple after-use separation of the materials.

Yeasts, which belong to the fungus kingdom, are relatively large eukaryotic and single-celled microorganisms (diameters typically measuring 2.0–4.0 µm). Their cell wall includes glucan, mannan, chitin protein, and a small amount of lipids, and it has many surface chemical groups such as carboxyl (–COOH), carbonyl (–C=O), amino (–NH_2_), hydroxyl (–OH), and phosphoryl (–P=O) groups. Moreover, yeast is very cheap, easy to obtain, and environmentally friendly. These advantages make yeasts appropriate and widely used as supports for bio-nanocomposites [31].

In the above-described context, the objectives of this study were to: (1) Prepare a bio-nanocomposite of yeast-Fe_3_O_4_ (magnetic yeast, MY) using an in situ one-step preparation of nano-Fe_3_O_4_; (2) use MY as the core to synthesize magnetic yeast-molecularly imprinted polymers (MY@MIPs) by a surface-imprinted polymerization method with MIPs as the shell and SMX as the template molecule; (3) characterize the resulting materials by Fourier-transform infrared spectroscopy (FT-IR), a vibrating sample magnetometer (VSM), X-ray diffraction (XRD), thermogravimetric analysis (TGA), specific surface area (*S*_BET_) determination, and scanning electron microscopy (SEM); (4) test the removal performance of MY@MIPs toward SMX and compare it with those of MY and MY@NIPs (magnetic molecularly imprinted polymers without template); and (5) explore the selective sorption capacity of MY@MIP in a real complex matrix (wastewater collected at a STP) and in the presence of other pharmaceuticals (diclofenac and carbamazepine).

## 2. Materials and Methods 

### 2.1. Chemicals and Materials 

Yeast cells (CICC 30225) were obtained from the China Center of Industrial Culture Collection (CICC). Iron salts used to produce MY were ferric chloride hexahydrate (FeCl_3_·6H_2_O) and ferrous chloride tetrahydrate (FeCl_2_·4H_2_O), purchased from Sigma-Aldrich (Stenheim, Germany). In addition, 2-vinyl pyridine (2-vpy), ethylene glycol dimethacrylate (EGDMA), acetonitrile (ACN), and azo-bis-isobutyronitrile (AIBN), which were also purchased from Sigma-Aldrich (Stenheim, Germany), were used for MIT. Other reagents used in this work included ammonium hydroxide, toluene (99.8%, Aldrich), ethanol (99.9%, Riedel-de Haën), methanol (99.99%, Fischer Chemical), and acetic acid (p.a., Merck). Ultrapure water was obtained from a Milli-Q water purification system (Millipore). SMX was purchased from TCI Europe (>98%); carbamazepine (CBZ; Sigma-Aldrich, 99%); diclofenac (DCF, TCI Europe, >98%). All solutions were stored at 4 °C immediately after preparation.

### 2.2. Materials Preparation

#### 2.2.1. Preparation of Magnetic Yeast (MY)

Nano-Fe_3_O_4_ was loaded onto the yeast cell surface by a one-step method as described by Tian et al. [32]. Briefly, the yeast cells were cultured in ultrapure water with glucose. After reaching the exponential growth phase (6–10 h), the yeast cells were collected by centrifugation (4000 rpm). Then, collected cells (1.0 g) were suspended in 40 mL of 0.125 M FeCl_3_ solution in a three-necked flask and stirred for 1 h at room temperature. After that, 0.6 g of FeCl_2_·4H_2_O was added under nitrogen atmosphere and stirred for another 1 h. The mixture was then heated in a water bath at 80 °C for 15 min, and the pH was adjusted to approximately 11 with 25% (*w/v*) ammonium hydroxide. Stirring was kept for 30 min and then stopped to age for 1 h. The resulting magnetic yeast (MY) was then washed, separated by applying a magnetic field, and then dried in an oven (35 °C, 4 h).

#### 2.2.2. Preparation of Magnetic Yeast-Based Molecularly Imprinted Polymer (MY@MIPs)

MY was treated as the core and the MIPs as the shell. The process used for the production of MY@MIPs was as follows: 1 mg of SMX (template molecule) and 4 mmol of 2-vpy (monomer) were dissolved in 60 mL of ACN/toluene (3/1; *v/v*). This solution was then self-polymerized for 8 h at room temperature (25 °C). Subsequently, 100 mg of MY (polymer supporter), 0.36 mmol of AIBN (initiator), and EGDMA (crosslinker) were added into the polymerized solution (template:monomer:crosslinker, 1:4:20), which was ultrasonicated for 10 min. The mixture was heated and maintained at 60 °C for 24 h under stirring with nitrogen protection. At last, the MY@MIPs were washed with methanol/formic acid (9/1; *v/v*) for 12 h and purified for 24 h by a Soxhlet extraction method (the extraction solution was methanol). Meanwhile, the MY@NIPs were also produced by following the above-described procedure but in the absence of the template.

### 2.3. Characterization of MY, MY@MIPs, and MY@NIPs

Fourier-transform infrared spectra of the produced materials were obtained in a Shimadzu-IRaffinity-1 equipment, using an ATR module (FTIR-ATR), under a nitrogen purge. The measurements were recorded in the range 500–4000 cm^−1^, 4.0 of resolution, 256 scans, and applying atmosphere and background correction.

A vibrating sample magnetometer (VSM EV9) with an oscillatory applied magnetic field (*H*) to a maximum of 22 kOe was used to determine the saturation magnetization (*M*_S_). The *M*_S_ was calculated by plotting the magnetic moment versus the applied magnetic field, and it corresponded to the plateau value of the magnetic moment reached divided by the sample mass (10 mg). The sample was encapsulated in an acrylic cylindrical container (5.85 mm of diameter and 2.60 mm of height), which was coupled to the lineal motor of the VSM EV9 instrument, centered between the two polar heads of the electromagnet used to fluctuate the magnetic field. The instrument was calibrated with a disk of pure nickel (8 mm of diameter) using a procedure that establishes the determination of the magnetic field, applied at around 1 Oe, while the dispersion of the magnetic moment is inferior to 0.5%.

X-ray diffraction (XRD, 5–90°) was measured on a D8-Focus X-ray diffractometer (Bruker Optics) with a test rate of 10°·min^-1^. The results were analyzed by Jade program (9.0) and Origin (9.0).

Thermogravimetric analysis (TGA) was performed in a thermogravimetric balance Setsys Evolution 1750, Setaram, TGA mode (S type sensor). The samples were heated at a heating rate of 10 °C min^−1^, under nitrogen atmosphere, from room temperature to 105 °C and from 105 °C to 900 °C, maintaining constant temperature until total stabilization of the sample mass at the end of both stages (approximately 30 min).

The *S*_BET_ and micropore volume (*W*_0_) were determined by nitrogen adsorption isotherms, acquired at 77 K using a Micromeritics Instrument, Gemini VII 2380, after outgassing the materials overnight at 120 °C. *S*_BET_ was calculated from the Brunauer–Emmett–Teller equation in the relative pressure range 0.01–0.1. Pore volume (*V*_p_) was estimated from the amount of nitrogen adsorbed at a relative pressure of 0.99.

The surface morphology of the materials was analyzed by scanning electron microscopy (SEM) using a Hitachi S4100. The images were obtained at magnifications of 500, 3000, and 10,000×.

### 2.4. Adsorptive Removal of SMX by the Produced Materials

The produced materials (MY, MY@MIPs, MY@NIPs) were used as adsorbents for the removal of SMX under batch operation conditions. Summarizing, the materials were put in contact with a 5 mg L^−1^ SMX solution in polypropylene tubes, which were shaken in a head-over-head shaker (80 rpm) for a predetermined period of time at controlled temperature (32 °C). The corresponding adsorbent material was separated from the suspension liquid by an external magnetic field. At last, the concentration of SMX in the liquid phase was measured by micellar electrokinetic chromatography (MEKC), using a methodology adapted from Silva et al. (2019) [33]. The experiments were conducted in triplicate, and control experiments without adsorbent were run in parallel. The performance of the materials was evaluated by carrying out kinetic, equilibrium, pH, selectivity, and regeneration/reutilization studies, described in detail in the next subsections.

#### 2.4.1. Kinetic Adsorption Studies in Ultrapure Water

In the kinetic study, tubes containing 250 mg of adsorbent material (MY, MY@NIPs, or MY@MIPs), together with 10 mL of a 5 mg L^−1^ SMX solution in ultrapure water, were incubated and shaken as described above. After shaking during defined periods of time (*t*, min), at intervals from 0 to 24 h, the materials were separated from the aqueous phase and the remaining SMX concentration in solution was measured by MEKC. At each time, the corresponding value of the adsorbed concentration (*q*_t_, mg·g^−1^) was determined as follows:

(1) $$q_{t}=\frac{C_{0}-C_{t}}{C_{m}}$$

where *C*_t_ (mg L^−^^1^) is the residual SMX concentration at time *t*, *C*_0_ is the initial SMX concentration (mg L^−^^1^), and *C*_m_ is the adsorbent dosage (mg·L^−^^1^).

When adsorption equilibrium was attained, the percentage of adsorption R (%) was determined as:

(2) $$R\;\left(\%\right)=\frac{C_{0}-C_{e}}{C_{0}}\times 100\%$$

where *C*_e_ (mg·L^−1^) is the residual SMX concentration at equilibrium.

#### 2.4.2. Equilibrium Adsorption Studies in Ultrapure Water

For the equilibrium studies, the corresponding adsorbent material (MY, MY@NIPs, or MY@MIPs), with doses ranging from 50 to 2000 mg L^−1^, was added to 10 mL of a 5 mg L^−1^ solution of SMX in ultrapure water. Tubes with the mixtures were shaken for 16 h, which allowed equilibrium to be reached. The materials were recovered from the suspension by the application of a magnetic field and the residual concentration of SMX was determined by MEKC. Then, for the different doses of material, the adsorbed concentration at the equilibrium (*q*_e_, mg·g^−1^) was determined as follows:

(3) $$q_{e}=\frac{C_{0}-C_{e}}{C_{m}}$$

where *C*_e_ (mg L^−^^1^) is the SMX concentration in the liquid phase at equilibrium.

### 2.5. Adsorptive Performance of MY@MIPs

From the results of the above-mentioned kinetic and equilibrium studies in ultra-pure water, the most efficient material for removal of SMX was MY@MIPs. Thus, in order to assess the practical application of this material, further studies were carried out on the adsorptive performance of MY@MIPs under different experimental conditions.

#### 2.5.1. Kinetic and Equilibrium Adsorption Studies in STP Effluent

The kinetic and equilibrium procedures described in Section 2.4. were carried out using MY@MIPs for the adsorptive removal of SMX from a real matrix, namely the effluent from a STP. In this case, 5 mg L^−1^ solutions of SMX were prepared using a STP effluent instead of ultrapure water. The effluent was collected from an urban STP in Aveiro (Portugal) that is designed to serve 159,700 population equivalents. This STP consists of primary and biological treatment stages. For this work, water was collected at the outlet of the biological decanter, as this is the final treated effluent that is discharged from the STP into the aquatic environment. Immediately after collection, the effluent was filtered through 0.45 μm, 293 mm Supor^®^ membrane disk filters (Gelman Sciences) and stored at 4 °C until use, which occurred within a maximum of 15 days. The collected effluent had a pH of 7.99, conductivity of 3.03 mS cm^−1^, and total organic carbon content of 21.5 mg L^−1^.

#### 2.5.2. pH Study

Adsorption studies on the effect of pH were carried out at 32 °C with the initial conditions of *C*_0_ = 5 mg L^−1^ in ultrapure water and *C*_m_ = 300 mg L^−1^. Experiments were carried out at three different pHs, namely 4, 7, and 8 (pH was adjusted by adding HCl or NaOH, 1 M). After shaking during 16 h, MY@MIPs were separated from the liquid suspensions, the residual concentration of SMX was analyzed by MEKC, and the corresponding *q*_e_ (mg g^−1^) at each pH was determined using Equation (3). 

#### 2.5.3. Selective Adsorption 

To study the selective capacity of MY@MIPs toward SMX, diclofenac (DCF) and carbamazepine (CBZ) were used as competing species in the adsorption experiments. These pharmaceuticals were selected due to their high global frequency of occurrence in wastewater, surface water, and groundwater and their recalcitrant properties, with low removal rates after conventional STP treatments [34]. The concentration of DCF and CBZ in ultrapure water solution was the same as that of SMX (5 mg L^−1^), the *C*_m_ was 300 mg L^−1^, the incubation temperature was 32 °C, the pH was 4, and shaking was maintained during 16 h. Then, the residual concentration of SMX at equilibrium was analyzed and the corresponding *q*_e_ (mg g^−1^) was determined with Equation (3).

#### 2.5.4. Regeneration and Reutilization

In order to evaluate the adsorptive performance after regeneration, after SMX saturation in ultrapure water, MY@MIPs were regenerated and then tested for the adsorption of SMX in four subsequent cycles. For the regeneration, saturated MY@MIPs were washed by methanol/acetic acid (9/1, *v/v*) through Soxhlet extraction during 72 h. Then, the regenerated material was used in adsorption experiments as described in previous sections (shaking during 16 h at 32 °C with the initial conditions of *C*_0_ = 5 mg L^−1^ in ultrapure water and *C*_m_ = 300 mg L^−1^). The residual concentration of SMX at the equilibrium was analyzed and the corresponding R (%) was determined as for Equation (2).

## 3. Results

### 3.1. Preparation of MY

In this study, an in situ one-step method was carried out to load nano-Fe_3_O_4_ particles on the surface of yeast, which was used as a biological solid support. Under alkaline conditions, Fe^2+^ and Fe^3+^ co-precipitated on the surface of yeast and then Fe(OH)_2_ or Fe(OH)_3_ was converted to nano-Fe_3_O_4_ at 80 °C, according to the following chemical reactions:

Fe^2+^ + 2OH^−^ → Fe(OH)_2_↓

Fe^3+^ + 3OH^−^ → Fe(OH)_3_↓

$$Fe{\left(OH\right)}_{2}+2Fe{\left(OH\right)}_{3}\overset{80\;{}^{\circ}C}{\to }Fe\left(Fe_{2}O_{4}\right)↓+4H_{2}O$$

**Figure 1:**
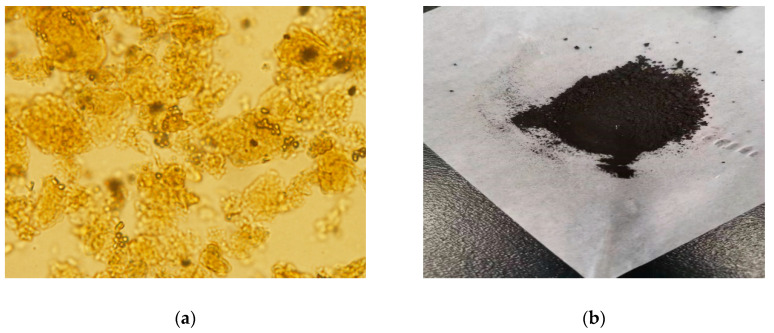
Optical microscopy photograph (500×) of magnetic yeast (MY) (**a**); actual size photograph of MY (**b**).

The observation of MY by a high-power optical microscope (Olympus CX22, Japan) clearly showed the loading of magnetic nanoparticles over yeast cells, as shown in Figure 1a. Meanwhile, Figure 1b represents the picture of MY at the actual size. Compared to other methods used to anchor Fe_3_O_4_ nanoparticles on the surface of yeast biomass, such as cross-linking or electrostatic-interaction-driven hypercoagulation, the one-step method applied here only took approximately 3.5 h, in opposition to the referred methods, which can take up to 13.5 and 6 h, respectively [35], without considering the time of washing and drying. Hence, the results suggest that the one-step method is an interesting synthesis option. 

### 3.2. Preparation of MY@MIPs

MY was used as support of a MIP-based material for the selective adsorption of SMX. Compared to other support materials such as SiO_2,_ carbon nanotubes, or Fe_3_O_4_-SiO_2_ used in the literature [36,37], MY particles can act as support without the need of an intermediate chemical modification step and can distinctly improve grafting efficiency. 

The shell of MIPs was co-polymerized on the surface of MY. Hence, to synthetize MIPs with affinity, selectivity, and appreciable removal capacity toward the target compound, the monomer and crosslinker types and the ratio of the reagents should be taken into account. Normally, if the template molecule has an alkaline chemical group, the monomer should be methacrylate (MAA), but if it has an acidic group, the monomer should be vinyl pyrimidine (vpy) [38]. As SMX has an oxazole moiety that displays acidity, 2-vpy was chosen as it has both a hydrogen-bond acceptor (N atom of pyridine) and alkalinity [39]. In this work, the molar ratio of the mixture of template and monomer was 1:4, as the monomer and template were in dynamic equilibrium, and it is not useful to add the monomer indiscriminately. Indeed, an excessive monomer may increase the non-selective sites, resulting in a selectivity decrease. On the other hand, during the synthesis of MIPs, in order to immobilize the template into the polymer without changing the spatial configuration of pores in the polymer, this must have a high rigidity. Therefore, it was necessary to use a crosslinker for increasing rigidity, EDGMA being selected due to its appropriate cost and solubility. However, if the ratio of monomer to crosslinker is too high, it will make the extraction of the template difficult due to the excessive rigidity of the MIP. Considering the referred considerations and conclusions from other studies [40,41], the ratio of monomer and crosslinker was selected to be 1:5.

### 3.3. Characterization of MY, MY@MIPs, and MY@NIPs

**Figure 2:**
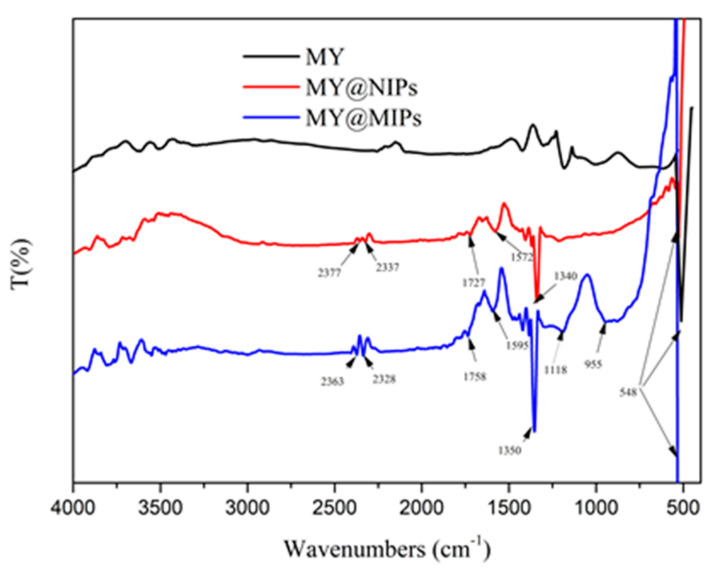
FTIR spectra of magnetic yeast (MY), magnetic yeast-molecularly imprinted polymers without template (MY@NIPs), and magnetic yeast-molecularly imprinted polymers (MY@MIPs).

FTIR spectra of the produced materials (MY, MY@MIPs, and MY@NIPs), which were obtained in order to shed some light about the chemical groups present on their surface, are depicted in Figure 2. At 548 cm^−1^, a characteristic adsorption peak belonging to the Fe-O chemical bond was observed for all materials. Compared to MY, MY@MIPs and MY@NIPs had some new peaks. Among them were the absorption bands at 2363 and 2328 cm^−1^ (MY@MIPs) and at 2377 and 2337 cm^−1^ (MY@NIPs), which were attributed to the stretching vibrations of -CN or -NC, respectively. The peak at 1758 cm^−1^ (MY@MIPs) or at 1727 cm^−1^ (MY@NIPs) belongs to the stretching vibration of C=O in the EGDMA ester group and the carboxyl group, suggesting that EGDMA worked on the surface. Moreover, MY@MIPs had new peaks at 1118 and 955 cm^−1^, which belonged to the symmetrical and asymmetric stretching vibration of C-O in EGDMA, respectively, and reflected that it had a cross-linking polymerization on the surface of MY@MIPs. In the spectra of MY@MIPs and MY@NIPs, adsorption peaks at 1350 or 1340 cm^−1^ were due to N–H bending vibrations, while this peak was very weak in MY, indicating the N–H bond of 2-vpy. Peaks at 1595 cm^−1^ (MY@MIPs) or 1572 cm^−1^ (MY@NIPs) were due to bending vibrations of N-H. 

The magnetic properties of the MY, MY@MIPs, and MY@NIPs were studied by VSM at room temperature, the *M*_S_ of each material being shown in Table 1. The *M*_S_ values were determined to be between 26 and 34 emu g^−1^, which were compatible with good magnetization. Indeed, Figure S1, within Supplementary Information, shows that MY@MIPs can be easily separated by an external magnetic field, which is beneficial for the after-use separation of the saturated MY@MIPs from treated water, achieving one of the major goals of this study.

**Figure 3:**
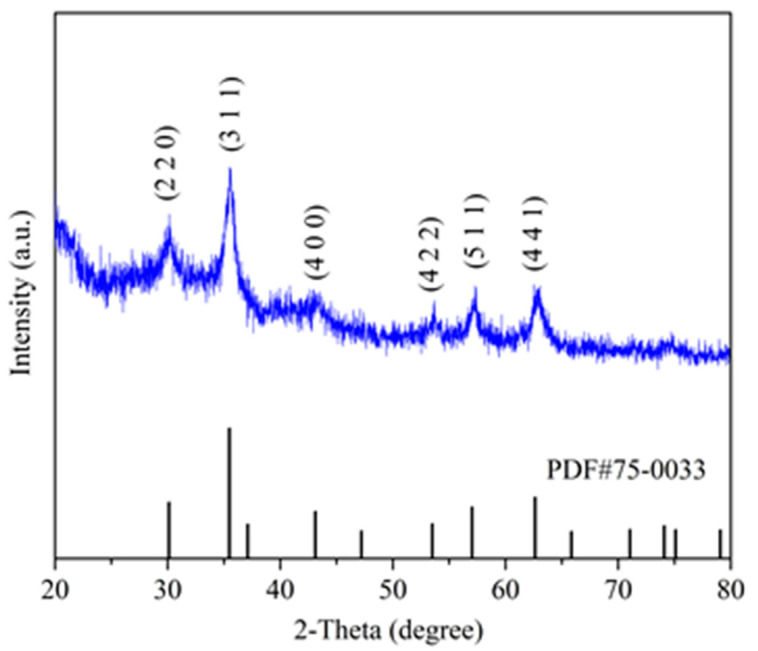
X-ray diffraction spectrum of MY.

The XRD spectrum of MY in the 2θ range of 20 to 80° is shown in Figure 3, where the (220), (311), (400), (422), (511), and (440) planes of Fe_3_O_4_ may be observed at 2θ = 30.22°, 35.40°, 43.36°, 53.68°, 57.21°, and 62.43°. This pattern is consistent with the standard XRD data of Fe_3_O_4_ in the JCPDS-International Centre for Diffraction Data (JCPDS Card: PDF#75-0033). Therefore, XRD results evidenced that Fe_3_O_4_ was successfully loaded onto the yeast surface during the production of MY and that, subsequently, surface molecular imprinting did not change the crystalline structure of magnetic nanoparticles. Similar patterns confirming the effective loading of magnetite have been reported in the literature on magnetic MIPs (MMIPs), including MMIPs produced for melamine analysis in milk [42], PEGylated magnetic core−shell structure-molecularly imprinted polymers (PMMIPs) for the specific adsorption of bovine serum albumin (BSA) [43], or core−shell MMIPs for the selective adsorption of tetracycline [44].

**Figure 4:**
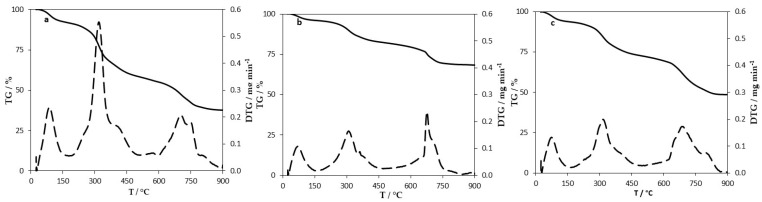
TG (full line) and DTG (dashed lines) curves of MY (**a**), MY@NIPs (**b**), and MY@MIPs (**c**).

The thermogravimetric (TG) and derivative thermogravimetric (DTG) curves of MY, MY@NIPs, and MY@MIPs are shown in Figure 4. All the materials evidenced three main weight loss peaks: The first at ~100 °C related to moisture; the second at ~300 °C related to the most thermolabile organic fraction; and the third centered at ~700 °C related to less thermolabile organic or inorganic fractions. For MY, a weight loss of approximately 62% was reached at 900 °C (Figure 4a); the second weight loss peak is particularly accentuated in this material, as it is the one with the highest amount of yeast per unit mass of material and, thus, yeast cells carbonized with increasing temperature. Meanwhile, MY@NIPs (Figure 4b) and MY@MIPs (Figure 4c) suffered, globally, a lower weight loss than MY, reaching 30% and 50% of weight loss, respectively, at 900 °C. This might be due to the introduction of less thermolabile structures in the composition of these materials (such as the magnetic nanoparticles and polymers).

The results of *S*_BET_ are shown in Table 1. The *S*_BET_ of each material was as follows: MY—38.8 m^2^ g^−1^, MY@MIPs—43.2 m^2^ g^−1^, and MY@NIPs—39.2 m^2^ g^−1^. Similar *S*_BET_ (47 m^2^ g^−1^) were determined for magnetic sorbents with a metal–organic framework core and MIP shell [45]. Meanwhile, lower *S*_BET_, between 6 and 11 m^2^ g^−1^, have been measured for magnetic sorbents based on the iron oxide (Fe_3_O_4_) core and MIP shell [46,47]. Regarding the average pore diameter (*D*), it was 5.71, 5.41, and 5.06 nm respectively for MY, MY@MIPs, and MY@NIPs. Therefore, the three produced materials are mesoporous with no significant differences between them in terms of porosity. 

**Figure 5:**
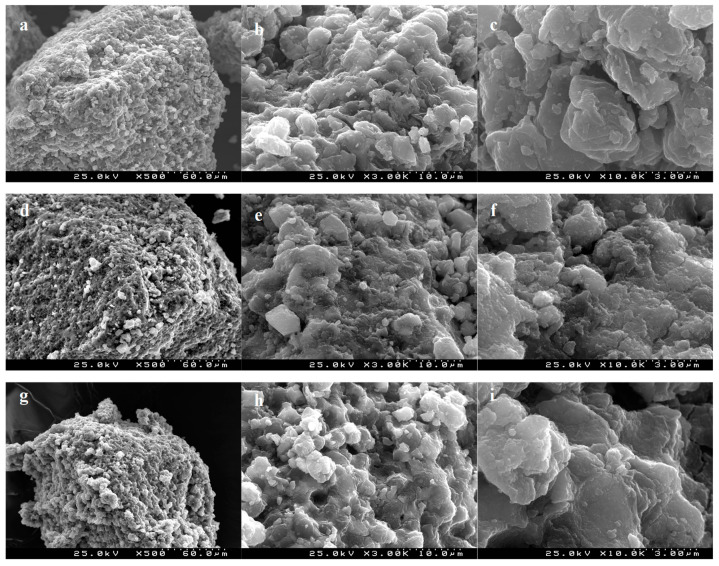
Scanning electron microscopy (SEM) images of MY (**a**–**c**), MY@NIPs (**d**–**f**), and MY@MIPs (**g**–**i**).

The surface of MY, MY@MIPs, and MY@NIPs was examined by SEM (Figure 5). All the figures suggested that the particles (either MY, MY@NIPs, or MY@MIPs) were elliptical, which is due to the use of yeast as support, as it has been observed to have an ellipsoid shape with uniform size [48]. As it may be seen, MY@NIPs and MY@MIPs have a dispersed and comparatively smoother appearance than MY, which is rough-faced due to the magnetic nanoparticles coating the smooth-faced yeast [49]. Moreover, under 3000× magnification, results showed that MY@MIPs had a better dispersion compared to MY and MY@NIPs. Under 10,000×, MY@MIPs showed a bigger porosity than the other materials, which may benefit the adsorption of SMX and improve the mass transfer rate from the aqueous phase.

Globally, the characterization results demonstrated the successful loading of Fe_3_O_4_ on the yeast surface and the preparation of MIPs on the surface of MY. 

### 3.4. Adsorptive Removal of SMX by the Produced Materials

#### 3.4.1. Adsorption Kinetics

**Figure 6:**
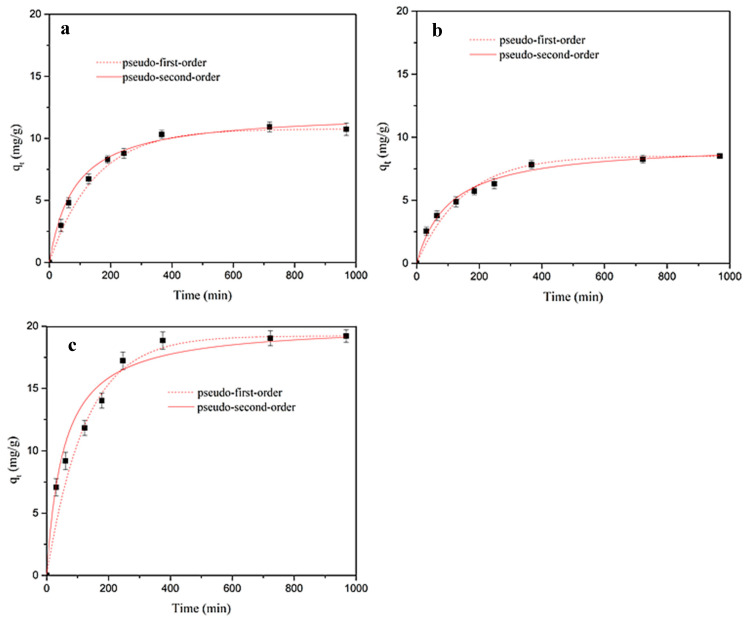
Experimental kinetic results together with pseudo-first- and pseudo-second-order model fittings for the adsorption of sulfamethoxazole (SMX) onto MY (**a**), MY@NIPs (**b**), and MY@MIPs (**c**) in ultrapure water.

The kinetic results on the adsorption of SMX onto the produced materials are shown in Figure 6, which evidences that, in all cases, the adsorbed concentration *q*_t_ (mg g^−1^) rapidly increased until 360 min of contact and then slowly increased until becoming stable. Moreover, all the materials performed quite similarly from a kinetic point of view.

Comparing the results obtained here to those reported in the literature, it may be said that a shorter equilibrium time (around 20 min, at room temperature) was determined for the adsorption of SMX onto core−shell MIPs on the surface of magnetic carbon nanotubes (MCNTs@MIP) by Zhao et al. [25]. Meanwhile, using MIPs on the surface of yeast (yeast@MIPs), Wang et al. [16] found that (at 298 to 318 K) 200 min were necessary to attain equilibrium for the adsorption of ciprofloxacin (CIP), and Pan et al. [50] observed an equilibrium time around 375 min for the adsorption (at 303 K) of cephalexin. In any case, it has been noticed that surface-imprinting improves the binding kinetics as compared to traditionally imprinted materials, which take longer (usually around 12–24 h) to attain adsorption equilibrium [51].

Pseudo-first-order [52] and pseudo-second-order [53] kinetic models were applied to describe the adsorption kinetics of SMX onto the produced materials. The formulation of the models is as follows:

Pseudo-first-order

(4) $$q_{t}=q_{e}\times \left(1-e^{\left(-k_{1}t\right)}\right)$$

Pseudo-second-order

(5) $$q_{t}=\frac{k_{2}\times q_{e}^{2}\times t\;}{1+k_{2}\times q_{e}\times t}$$

where *k*_1_ (min^−1^) and *k*_2_ (g mg^−1^ min^−1^) are the pseudo-first-order and the pseudo-second-order rate constants.

The non-linear fitting kinetic parameters are summarized in Table 2. According to the correlation coefficient (R^2^) and concordance between experimental and fitted *q*_e_ values, both models described the SMX adsorption onto the produced materials, with the pseudo-second-order model describing slightly better the results onto MY and MY@NIPs and the pseudo-first-order model onto MY@MIPs.

#### 3.4.2. Adsorption Isotherm

**Figure 7:**
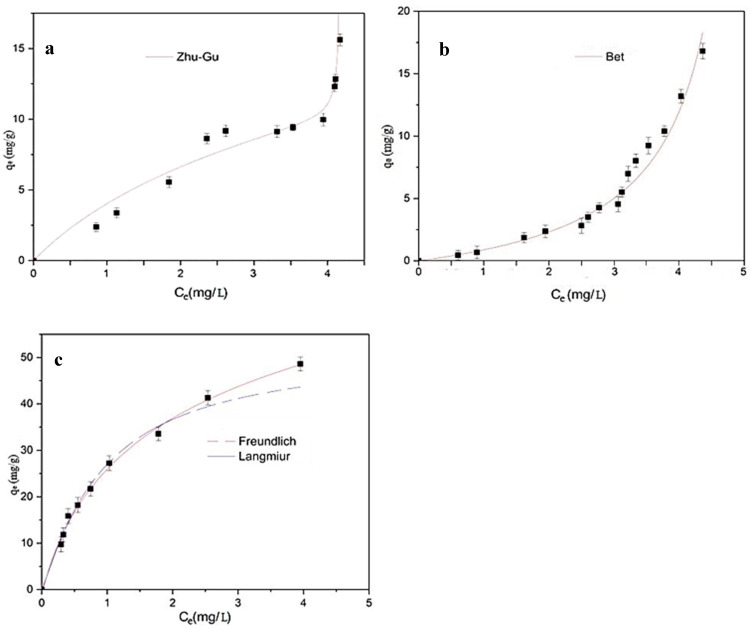
Experimental equilibrium results together with fittings to the considered models for the adsorption isotherm of SMX onto MY (**a**), MY@NIPs (**b**), and MY@MIPs (**c**) in ultrapure water.

Equilibrium results on the adsorption of SMX onto the produced materials are shown in Figure 7. With the aim of describing these results, four isotherm models were used: Langmuir [54] and Freundlich [55] isotherm models for the adsorption of SMX onto MY@MIPs; BET isotherm [56] for the adsorption onto MY@NIPs; and Zhu−Gu isotherm [57] for the adsorption onto MY. The equations of these models are as follows:

Langmuir isotherm

(6) $$q_{e}=\frac{q_{m}\times b\times C_{e}}{1+b\times C_{e}}$$

Freundlich isotherm

(7) $$q_{e}=k_{f}\times {c_{e}}^{\frac{1}{n}}$$

BET isotherm

(8) $$q_{e}=\frac{q_{m}\times c\times C_{e}}{\left(1-c\times C_{e}\right)\times \left(1-c\times C_{e}+c\times C_{e}\right)}$$

Zhu−Gu isotherm

(9) qe=qm×(g×Ce×(1r+e×Cer−1))(1+g×Ce)×(1+e×Cer−1)

where *q*_m_ is the maximum adsorption capacity (mg g^−1^); *b* (L mg^−1^) is the Langmuir equilibrium constant; *k*_f_ (mg g^−1^ (mg L^−1^)^−1/n^) is the Freundlich constant; *n* is the degree of non-linearity in the Freundlich isotherm; *c* is the BET constant, related to the energy of adsorption in the first adsorbed layer; *g* is the Zhu−Gu constant related to the first adsorption step (the first layer of molecules on the materials); *e* is the Zhu−Gu constant related to the subsequent layers adsorbed; and *r* is the aggregation number in the Zhu−Gu isotherm.

Experimental results on the equilibrium of SMX adsorption onto the produced materials are depicted in Figure 7 together with fittings to the above-mentioned isotherm models. From Figure 7, it is evident that, contrarily to the adsorption onto MY@MIPs, in the case of MY and MY@NIPs, the *q*_e_ did not tend to stabilization. Furthermore, in the *C*_e_ range between 0 and 3 mg L^−1^, a lower *q*_e_ occurred for MY@NIPs than for MY. This may be related to the presence of chemical groups on the surface of MY, which were able to bind SMX groups, but became inaccessible in MY@NIPs due to molecular imprinting. In addition, a first stage with stabilization of *q*_e_ at *C*_e_ around 3 mg L^−1^ may be observed in the MY isotherm, which could be associated with the saturation of the chemical adsorption sites. In the case of MY@MIPs, the isotherm showed an increase in *q*_e_ with *C*_e_ with a stabilization trend from *C*_e_ ~ 3 mg L^−1^. Furthermore, it should be noted that, at relatively low *C*_e_, the *q*_e_ values determined for MY@MIPs are higher than those for MY and MY@NIPs, which points to their larger affinity for SMX.

The fitted equilibrium parameters are shown in Table 3 together with the correlation coefficients of the fittings (*R^2^*). In the case of SMX adsorption onto MY@MIPs, the Langmuir isotherm provided the best fitting of equilibrium results with a higher R^2^ than the Freundlich model. From the results of non-linear fittings for the different isotherm models, the values of *q*_m_ for MY, MY@NIPs, and MY@MIPs were, respectively, 23 ± 1, 3.8 ± 0.3, and 77 ± 3 mg g^−1^. These values indicate that molecularly imprinted polymers with the template resulted in a substantial increase in the monolayer adsorption capacity, SMX adsorbing onto the surface of MY@MIPs in a homogeneous distribution by occupying specific sites. Similarly, equilibrium results on the adsorption of SMX onto the MCNTs@MIP produced by Zhao et al. [25] also fitted the Langmuir isotherm, but with a considerably lower *q*_m_ (0.87 mg g^−1^). Indeed, compared to other materials used for the adsorption of SMX (Table 4), MY@MIPs are competitive in terms of SMX adsorption capacity. 

#### 3.4.3. Kinetic and Equilibrium Adsorption Studies from STP Effluent

**Figure 8:**
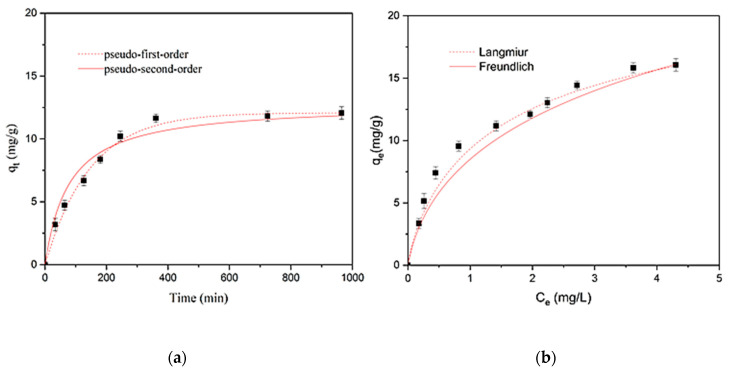
Experimental results together with fittings to the considered models for the adsorption kinetics (**a**) and adsorption equilibrium isotherm (**b**) of SMX onto MY@MIPs in sewage treatment plant (STP) effluent.

In order to assess the practical applicability of MY@MIPs, kinetic and equilibrium experiments were carried out in a real matrix, namely the effluent from a STP. The obtained results together with fittings to the considered kinetic and equilibrium models are in shown in Figure 8, and the fitted parameters are depicted in Table 5. As it may be seen, the pseudo-second-order and the Langmuir isotherm models were those that best described the kinetic and equilibrium experimental results, respectively. On the other hand, it is evident in Figure 8 that, under identical experimental conditions, the adsorption velocity was slower and the *q*_e_ values were lower for the STP effluent than they were for ultrapure water. This was confirmed by the parameters in Table 5, especially by the comparatively lower *q*_m_ (24 ± 2 mg g^−1^) than in ultrapure water, which might be related to interferences due to the complex composition of the STP effluent. 

### 3.5. pH Study

**Figure 9:**
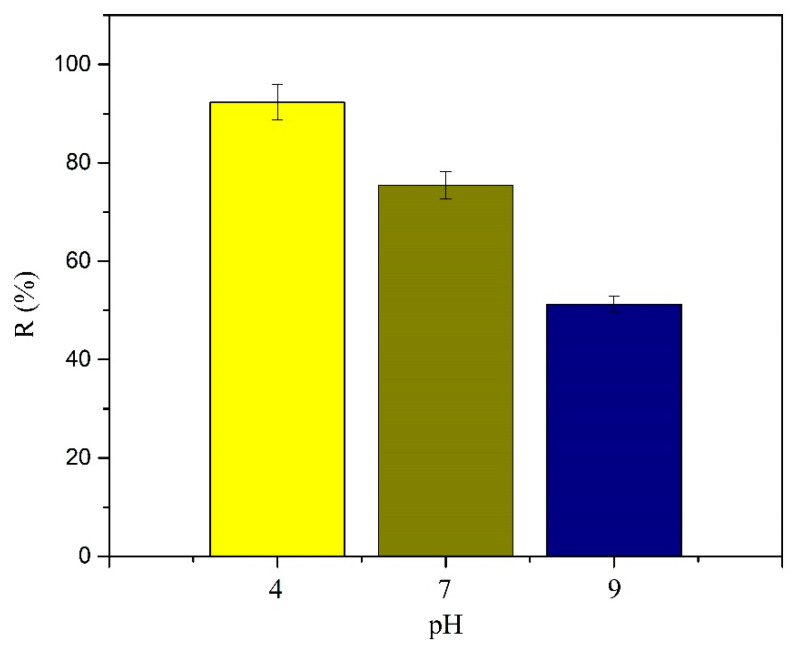
Effect of pH on the percentage of SMX adsorption (R (%)) onto MY@MIPs.

Results from the study of pH effects on the adsorption of SMX onto MY@MIPs are shown in Figure 9. Under identical experimental conditions, except for the pH, decreasing *q*_e_ values were obtained at pH 4 > pH 7 > pH 9, thus indicating that SMX adsorption onto MY@MIPs was favored under acidic conditions. This may be related to the pH influence on the status of not only the adsorbate (by protonation/deprotonation) but also the adsorbent (by surface charge). For SMX, the p*K*_a_ values are 1.97 and 6.16 (Table S1, as Supplementary Information), which means that SMX is mostly positively charged (protonated NH_2_ groups, NH_3_^+^ groups) at pH < 1.97 but predominantly negatively charged (deprotonated NH groups, N−) at pH > 6.16. Therefore, at the experimental pH 4, adsorption of SMX in the non-ionic form was favored, while at pH 7 and 9, SMX was mostly present in the anionic form, which partially hindered its adsorption. Indeed, the decrease in SMX adsorption from wastewater (with pH > 7) has already been related to electrostatic repulsion between the negatively charged SMX and the negatively charged surface of the waste-based adsorbents [59]. Moreover, the monomer used in the synthesis of MIPs was 2-vpa and the p*K*_a_ of pyridine was 5.21, which is, therefore, negatively charged when pH > 5.21. Thus, at the experimental pH 7, electrostatic repulsion forces between SMX and MY@MIPs cannot be disregarded, these increasing at pH 9. 

### 3.6. Selective Adsorption

**Figure 10:**
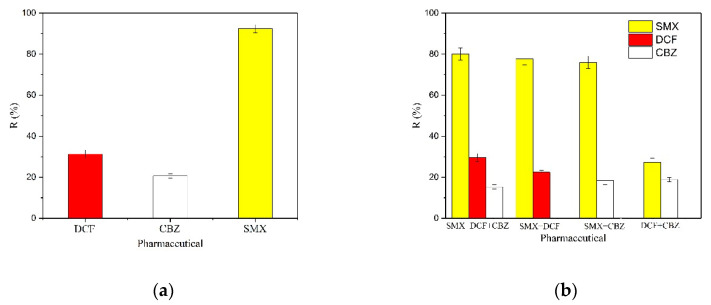
Percentage of adsorption (R (%)) of SMX, diclofenac (DCF), and carbamazepine (CBZ) onto MY@MIPs from single solution (**a**) and ternary solution (**b**).

In order to find out the selectivity of MY@MIPs toward SMX, its adsorption was compared to those of DCF and CBZ from their single solution and then from their ternary solution. The values of percentage of adsorption (R (%)) for the single adsorption of each pharmaceutical are shown in Figure 10a and for adsorption from their ternary solution in Figure 10b.

The results in Figure 10a evidence that MY@MIPs have a larger R (%) for SMX than for DCF or CBZ. Furthermore, under identical experimental conditions but from the ternary solution of the considered pharmaceuticals (Figure 10b), selective adsorption of SMX onto MY@MIPs occurred. Indeed, the R (%) determined for SMX from the ternary solution was just slightly lower than from its single solution, which points to the selectivity of MY@MIPs. Moreover, the results reflected that the adsorption of MY@MIPs was SMX > DCF > CBZ.

In this work, 2-vpy was the monomer and it was combined through –NH_2_. Moreover, considering the structure and properties of SMX, DCF, and CBZ, which are depicted in Table S1, they all have –NH_2_ and/or –NH groups. However, SMX has two amino groups: -NH and –NH_2_, which is probably the main reason for its selective adsorption onto MY@MIPs under the presence of DCF and CBZ. For DCF and CBZ, the p*Ka* was 4 and 15.96 (Table S1), respectively. Meanwhile, the p*K*_a_ value of 2-vpy (monomer) is 5.21, which may explain why MY@MIPs had a better removal ability for DCF than for CBZ. Selectivity toward SMX was also verified by Zhao et al. [25], who prepared MCNTs@MIP by using SMX as the template molecule and copolymerization of vinyl end groups on the surface of MCNTs [25]. These authors demonstrated the selective adsorption of SMX under the presence of other sulfonamides (SAs), namely sulfamethazine (SMZ), sulfamerazine (SMR), sulfadimethoxine (SDM), and sulfameter (SME). Still, the adsorbed concentration of SMX from the quinary solution was lower than from its single solution, which was ascribed to the close structure of the other SAs, which, therefore, could competitively occupy the imprinted sites.

### 3.7. Regeneration and Reutilization

Saturation of the produced MY@MIPs with SMX was carried out as described in Section 2.4.1. At that moment, R (%) calculated by Equation (2) was 92 ± 4%. Then, saturated MY@MIPs was regenerated as indicated in Section 2.5.4. and reused for the adsorption of SMX until saturation. A total of four regeneration/reutilization cycles were performed and the R (%) calculated for each of them are shown in Table 6.

As it may be seen, after cycles 1 and 2, the R (%) values decreased to 73 ± 3 and 61 ± 2%, respectively. Such decreases (21 and 34%, respectively, in cycle 1 and 2) indicate that the regeneration procedure affected the adsorption sites on the surface of MY@MIPs, which lost efficiency in the removal of SMX. After cycle 2, just a slight decrease in R (%) occurred, its value being similar in cycles 3 and 4 (58 ± 1 and 55 ± 2%, respectively). Therefore, deterioration of MY@MIPs adsorptive properties was not progressive with successive regenerations but occurred initially, with the performance remaining stable after cycle 2. MIPs sorbents are known to be easily regenerated by washing with organic solvents, with mixtures of methanol and acetic acid having been successfully employed to remove adsorbed pharmaceuticals [60]. Using the same regeneration agent as in this work, namely methanol/acetic acid (9/1, *v/v*), Dai et al. [61] regenerated MIPs synthesized for the adsorption of diclofenac and carried out thirty cycles with ≥95% recovery. Likewise, Duan et al. [62] also used this mixture for the regeneration of a multitemplate MIP, which was used in twenty regeneration/reutilization cycles, giving ≥95% removal of ibuprofen, naproxen, ketoprofen, diclofenac, and clofibric acid. Wang et al. [48], who used MIPs on the surface of yeast (yeast@MIPs), desorbed ciprofloxacin using the same mixture with losses of only about 8.5% of initial capacity after five cycles. Therefore, the relatively larger deterioration of the adsorptive performance observed in the present work may be related to the fact that magnetic yeast was used here as MIPs support. Thus, further work is to be carried out on the regeneration of the produced MY@MIPs, to maintain a high R (%) upon cyclic operation.

## 4. Conclusions

This work developed an efficient strategy to prepare yeast-Fe_3_O_4_ (magnetic yeast, MY) and then used molecularly imprinted technology (MIT) to modify MY. The characterization of the produced magnetic yeast-molecularly imprinted polymers (MY@MIPs) showed that elliptical and monosized imprinted polymeric nanospheres with a surface area of about 43.2 m^2^ g^−1^ were successfully produced. Sulfamethoxazole (SMX) adsorption studies using MY@MIPs indicated that the equilibrium was attained in 360 min either in ultrapure water or in a sewage treatment plant (STP) effluent. The Langmuir isotherm model provided the best fitting of equilibrium results and pointed to the monolayer and favorable adsorption of SMX onto MY@MIPs. In addition, the fitted parameters of the Langmuir isotherm model indicated that the maximum SMX adsorption capacity of MY@MIPs was 77 and 24 mg g^−1^ in ultrapure water and STP effluent, respectively. The pH study pointed out that hydrogen binding was underneath the SMX adsorption onto MY@MIPs. Moreover, MY@MIPs showed successful selective adsorption of SMX from ternary solution under competition by other pharmaceuticals, namely diclofenac (DCF) and carbamazepine (CBZ). Finally, regeneration implied a reduction in SMX removal by MY@MIPs in the first two cycles, then tending to stabilization. Overall, it may be concluded that the MIPs-coated magnetic yeast designed here could be an alternative adsorbent for the selective removal of SMX from complex matrices such as wastewaters.

## Figures and Tables

**Table 1:** Physical characterization of the produced materials.

Materials	*S*_BET_ (m^2^ g^−1^)	*V*_p_ (cm^3^ g^−1^)	*D* (nm)	*M*_S_ (emu g^−1^)
MY	38.8	0.11	5.71	26.1
MY@NIPs	39.2	0.11	5.41	24.2
MY@MIPs	43.2	0.11	5.06	34.1

N_2_ adsorption at −196 °C; *V*_p_ = total pore volume; *D* = average pore diameter; *M*_S_ = saturation magnetization.

**Table 2:** Kinetic parameters corresponding to the adsorption of SMX onto MY, MY@NIPs, and MY@MIPs in ultrapure water.

Materials	Experimental	Pseudo-First Order Model			Pseudo-Second Order Model		
	*q*_e_ (mg g^−1^)	*q*_e_ (mg g^−1^)	*k_1_* (min^−1^)	R^2^	*q*_e_ (mg g^−1^)	*k_2_* (g mg^−1^ min^−1^)	R^2^
MY	10.6 ± 0.7	11.1 ± 0.9	0.993 ± 0.003	0.931	12.5 ± 0.8	0.009 ± 0.005	0.994
MY@NIPs	8.2 ± 0.2	8.1 ± 0.1	0.993 ± 0.002	0.978	9.5 ± 0.2	0.009 ± 0.005	0.988
MY@MIPs	19 ± 1	19 ± 1	0.992 ± 0.001	0.966	21 ± 1	0.001 ± 0.004	0.952

**Table 3:** Equilibrium parameters corresponding to the adsorption of SMX onto MY, MY@NIPs, and MY@MIPs from ultrapure water.

Materials	Isotherm Model	Parameters	Fitted Values
MY	Zhu−Gu	*q*_m_ (mg g^−1^)	23 ± 1
		*r*	0.834 ± 0.005
		*e*	0.775 ± 0.005
		R^2^	0.957
MY@NIPs	BET	*q*_m_ (mg g^−1^)	3.8 ± 0.3
		*c*	0.205 ± 0.008
		R^2^	0.979
MY@MIPs	Freundlich	*k*_f_ (mg g^−1^ (mg L^−1^)^−1/n^)	26 ± 1
		1/*n*	0.575 ± 0.003
		R^2^	0.965
	Langmuir	*q*_m_ (mg g^−1^)	77 ± 3
		*b* (L mg^−1^)	0.498 ± 0.003
		R^2^	0.998

**Table 4:** Maximum Langmuir adsorption capacities (*q*_m_, mg g^−1^) of different MIPs used for the adsorption of SMX.

Adsorbent (mg)	*q*_m_ (mg g^−1^)	Experimental Conditions	References
MIPs (100)	16.5	pH = 3; Time = 15 min; room temperature; *C*_SMX_ = 7500 μmol/L; *V* = 15 mL	[19]
Fe_3_O_4_-chitosan MIPs (10)	4.32	pH = 4; Time = 30 min; room temperature; *C*_SMX_ = 200 μg/mL; *V* = 10 mL	[5]
Magnetic carbon MIPs (15)	0.87	pH = 4; Time = 60 min; room temperature; *C*_SMX_ = 8 μg/mL; *V* = 20 mL	[25]
Monolithic MIPs (200)	0.02	pH = 3; Time = 30 min; room temperature; *C*_SMX_ = 4 μmol/L; *V* = 10 mL	[58]
MY@MIPs (250)	77	pH = 4; Time = 360 min; room temperature; *C*_SMX_ = 5 mg/L; *V* = 10 mL	This study

C_SMX_ = Initial concentration of SMX.

**Table 5:** Kinetic and equilibrium parameters corresponding to the adsorption of SMX onto MY@MIPs from the STP effluent.

Adsorption	Models	Parameters	Fitted Values	Experimental
Kinetics	Pseudo-first order	*q*_e_ (mg g^−1^)	11.9 ± 0.3	12.3 ± 0.2
		*k_1_* (min^−1^)	0.471 ± 0.004	
		R^2^	0.978	
	Pseudo-second order	*q*_e_ (mg g^−1^)	13.8 ± 0.7	12.3 ± 0.2
		*k_2_* (g mg^−1^ min^−1^)	0.009 ± 0.005	
		R^2^	0.979	
Equilibrium	Freundlich	*k*_f_ (mg g^−1^ (mg L^−1^)^−1/n^)	8.5 ± 0.4	
		1/*n*	0.387 ± 0.008	
		R^2^	0.979	
	Langmuir	*q*_m_ (mg g^−1^)	24 ± 2	
		*b* (L mg^−1^)	0.609 ± 0.004	
		R^2^	0.989	

**Table 6:** SMX adsorption onto MY@MIPs in subsequent cycles after regeneration.

Cycles	R (%)
SMX saturation	92 ± 4
1	73 ± 3
2	61 ± 2
3	58 ± 1
4	55 ± 2

## Supplementary Materials

The following are available online at https://www.mdpi.com/2073-4360/12/6/1385/s1, Table S1: Physico-chemical properties of the pharmaceuticals used in this study (Source: Drugbank), Figure S1: MY@MIPs in the presence (**a**) and absence (**b**) of an external magnetic field.
