# Supplementary material for: Core−Shell Molecularly Imprinted Polymers on Magnetic Yeast for the Removal of Sulfamethoxazole from Water
Source: Polymers (Basel). 2020 Jun 20;12(6):1385. doi: 10.3390/polym12061385 (PMC7362263; doi:10.3390/polym12061385)
Supplement: Supplementary file 1 [file polymers-12-01385-s001.pdf]

SUPPLEMENTARY INFORMATION

Core-shell molecularly imprinted polymers on magnetic yeast for the removal of sulfamethoxazole from water

Liang Qiu[1,2], Guilaine Jaria[2], María Victoria Gil[3], Jundong Feng[1], Yaodong Dai[1],Valdemar I. Esteves[2], Marta Otero[4,*], Vânia Calisto[2]

1 Department of Materials Science and Technology, Nanjing University of Aeronautics & Astronautics, Nanjing 210016, P.R. China; qiuliangjay@qq.com (L.Q.); jundongfeng@nuaa.edu.cn (J.F); yd_dai@nuaa.edu.cn (Y.D.)
2 CESAM & Department of Chemistry, University of Aveiro, Campus Universitário de Santiago, 3810-193 Aveiro, Portugal; jaria.guilaine@ua.pt (G.J.); valdemar@ua.pt (V.I.E.); vania.calisto@ua.pt (V.C)
3 Instituto de Ciencia y Tecnología del Carbono, INCAR-CSIC, Apartado 73, 33080 Oviedo, Spain; victoria.gil@incar.csic.es (M.V.G)
4 CESAM & Department of Environment and Planning, Campus Universitário de Santiago, 3810-193 Aveiro, Portugal; marta.otero@ua.pt (M.O.)
* Correspondence: marta.otero@ua.pt (M.O.)

Table S1. Physico-chemical properties of the pharmaceuticals used in this study (Source: Drugbank).

Pharmaceutical (formula)	Structure	Mw (g mol^{-1})	Sw (mg L^{-1})	pKa	Log K_{ow}	PSA (A^2)	HBAC
Sulfamethoxazole ($C_{10}H_{11}N_3O_3S$)	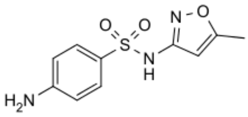	253.28	0.459	6.16 (strongest acidic)<br>1.97 (strongest basic)	0.79	98.22	4
Diclofenac ($C_{14}H_{11}Cl_2NO_2$)	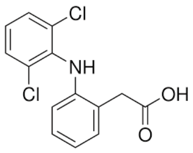	294.15	0.00447	4 (strongest acidic)<br>-2.1 (strongest basic)	4.98	49.33	3
Carbamazepine ($C_{15}H_{12}N_2O$)	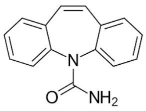	236.27	0.11	15.96 (strongest acidic)<br>-3.8 (strongest basic)	2.77	46.33	1

PSA= Polar Surface Area; HBAC = Hydrogen Bound Acceptor Count; Sw = water solubility (25ºC)

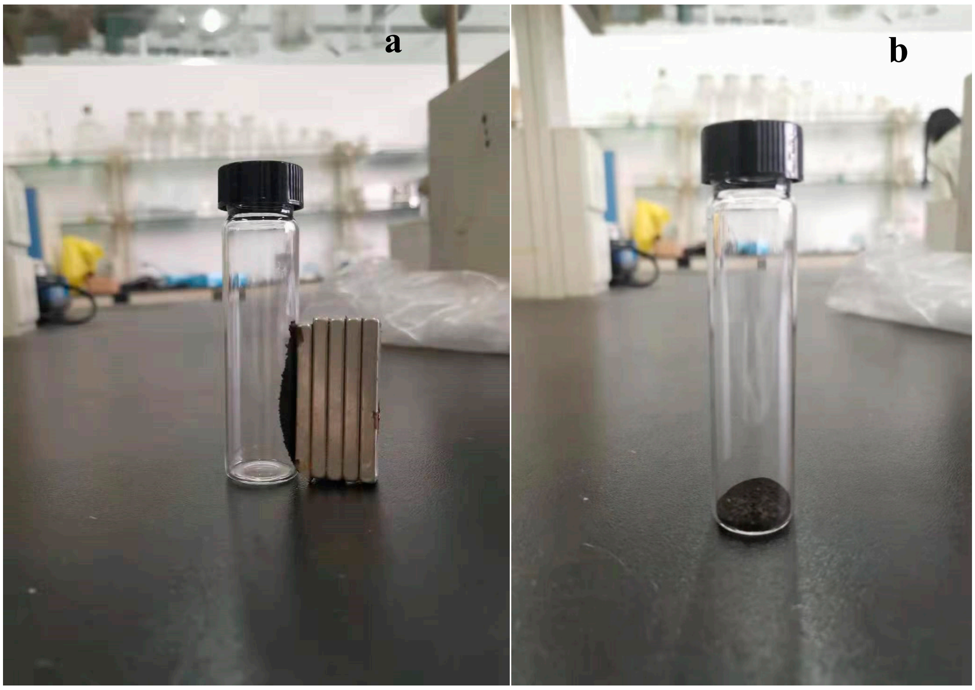

Fig. S1: MY@MIPs in the presence (a) and absence (b) of an external magnetic field.
